# Supplementary material for: Facility and care provider emergency preparedness for neonatal resuscitation in Kano, Nigeria
Source: PLoS One. 2022 Jan 7;17(1):e0262446. doi: 10.1371/journal.pone.0262446 (PMC8741031; doi:10.1371/journal.pone.0262446)
Supplement: S1 File — (PDF) [file pone.0262446.s001.pdf]

Facility and care provider emergency preparedness for neonatal resuscitation in Kano, Nigeria

Demographic Information

1. Age (years)____________________
2. Gender [ ] Male [ ] Female
3. Designation [ ] Doctor [ ] Nurse [ ] Midwife [ ] Others, specify……………
4. Health facility [ ] Primary [ ] Secondary [ ] Tertiary
5. Ward/Unit [ ] Neonatal Unit [ ] Labor/delivery room [ ] Operating theatre. [ ] Pediatric ward
6. Years of experience in work_______ and specified ward _________
7. Health Facility (Name, LGA) ______________________
8. Have you ever received training on neonatal resuscitation? [ ] Yes [ ]No

   If yes, when? [ ] <1yr [ ] 1-3yrs [ ] 4-6yrs [ ] 7-9yrs [ ] >10yrs
9. Number of deliveries (vaginal or CS) attended in the last year?

   [ ] None [ ] Less than 10 [ ] 10–19 [ ] 20-29 [ ] >30
10. Number of neonates resuscitated by you in the last year

    [ ] None [ ] 1-5 [ ] 6–10 [ ] 11-15 [ ] 15-20 [ ] >20
11. Approximate number of new-born deaths per month occurring within 24 hours after delivery ________
12. Approximate number of deliveries per month _______________
13. Total number of staff dedicated for neonatal care in your facility? ___________

Knowledge

Instruction: *Only one response is required for each question please*

1. What is the sequence of newborn resuscitation?

   [ ] Airway management, temperature control, circulation , drugs

   [ ] Circulation, temperature control, airway management, drugs

   [ ] Temperature control, airway management, circulation , drugs

   [ ] Drugs, temperature control, airway management, circulation

2. The most important and effective action in neonatal resuscitation is?

   [ ] Positioning the baby [ ]Suctioning [ ]Ventilating the lungs [ ]Drying and keeping warm

3. Initial steps for newborn resuscitation are to

   [ ]Provide warmth, , clear secretions if needed, dry and stimulate, head positioning

   [ ] Dry and stimulate, provide warmth, head positioning, clear secretions if needed

   [ ] Head positioning, clear secretions if needed, dry and stimulate, provide warmth

   [ ] Clear secretions always, dry and stimulate, provide warmth, head positioning

4. After the initial steps are completed, further decisions are based on assessment of?

   [ ] Breathing and heart rate. [ ] Appearance and breathing

   [ ] Temperature, appearance and breathing. [ ] Temperature, appearance, breathing and heart rate

5. What are the indications to ambu bag a new-born after delivery?

   [ ] All babies that didn't cry immediately after birth

   [ ] Apnoea, gasping , heart rate <100 beats per minute

   [ ] Secretions, laboured breathing, heart rate >100 beats per minute

   [ ] Laboured breathing or persistent cyanosis

6. Which baby should receive oxygen during resuscitation?

   [ ] All new-born babies that need ambu bagging

   [ ] Babies with laboured breathing or persistent cyanosis

   [ ] All babies that didn't cry immediately after birth

   [ ] Babies with fast breathing. [ ] Don't know

7. What is the targeted pre-ductal oxygen saturation at the fifth minute?

   [ ] 75-80% [ ] 80-85% [ ] 85%-95% [ ] 100% [ ]Don't know

8. The initial airway management of newborn not breathing is?

   [ ] Position ± suction nose then mouth and ambu bag [ ] Suction mouth then nose, position and ambu bag

   [ ] Ambu bag with chest compression [ ] Position ± suction mouth then nose and ambu bag

9. At what rate do you give breaths by bag and mask while resuscitating term neonates?

   [ ] 30-40/min [ ] 40–60/min [ ] 20–30/min [ ] >60/min

10. Chest compressions in infants are indicated when heart rate remains less than _______ bpm despite ___________ seconds of effective Positive Pressure Ventilation.

    [ ]100, 60 [ ] 60, 15 [ ] 100, 30 [ ] 60, 30

11. Adrenaline is indicated when heart rate remains less than _______ bpm despite ___________ seconds of effective compression and ventilation.

    [ ] 100, 60 [ ] 60, 15 [ ] 100, 30 [ ] 60, 60

12. The most sensitive indicator of adequate resuscitation is?

    [ ] increase in heart rate [ ] increase in respiratory rate [ ] increase in temperature [ ] increase in crying

Self-reported preparedness and attitude

Instruction: *For question 1-2 only, please respond using the scale below, tick correct response for questions 4*

1=Very poor 2=Poor 3=Average 4=Good 5=Very good 6=Excellent

1. What is your comfort level during newborn resuscitation? _________
2. What is your self-assessed knowledge on neonatal resuscitation? __________
3. What is your opinion on the general level of facility preparedness for new-born resuscitation? ____________
4. Do you often feel the need to call for help? [ ] Yes [ ] No

Practice

1. Do you use a partograph to assess if an unborn baby will require resuscitation? [ ] Yes [ ] No
2. How many staff participate during new-born resuscitation? __________
3. How do you maintain normal temperature in newborns? *(Multiple responses possible).*

   [ ] Clothing [ ] Radiant warmer [ ] Polythene bag [ ] Skin to skin [ ] Others, specify_____________
4. Do you insert umbilical catheters? [ ] Yes [ ] No.

   if yes, how often in a month [ ] 1-5 [ ] 6-10 [ ] >11-15 [ ] >15
5. What is the routine practice in your facility regarding cutting of the umbilical cord? [ ] Cord is cut immediately after the delivery of the baby [ ] Cord is cut after one minute of delivery of the baby [ ] Cord is cut after pulsations stop. [ ] Cord is cut after 5 minutes
6. What do you do when a term baby doesn't cry after birth?

   [ ] Provide warmth, position and ambu bag [ ] Tactile stimulation, rubbing the back and shaking the baby [ ] Wrap baby in mothers dry cloth and refer to nursery [ ] Sprinkle water

7. For term babies born through meconium-stained liquor, what do you do first?

   [ ] Intubate and suction. [ ] Suction the mouth then nose [ ] Suction the nose then mouth

   [ ] Stimulate and ventilate

8. For persistent apnea, just after birth, what would you do?
   [ ] Continue tactile stimulation a little bit more vigorously [ ] Give positive pressure ventilation promptly [ ] Give free flow oxygen

9. What chest compression/respiration ratio do you perform during cardiac compression?
   [ ] 1:1. [ ] 2:1 [ ] 3:1 [ ] 4:1

10. During chest compression how much pressure do you use?

    [ ] Depress the sternum to 1/3rd of AP diameter of chest

    [ ] Depress the sternum to 1/2 of AP diameter of chest

    [ ] There is no strict guideline; it varies depending upon the weight of the baby

    [ ] Increasing pressure till there is a response

11. Where do you apply pressure when performing chest compressions on the newborn?

    [ ] Lower third of the sternum [ ] Middle third of the sternum [ ] Upper third of the sternum [ ] Any of the above sites are acceptable.

12. During resuscitation of a newborn, despite adequate ventilation and chest compressions, the heart rate has not increased. What is the most appropriate next step in management?

    [ ] Administer epinephrine [ ] Continue chest compressions and ambu bagging

    [ ] Start dopamine infusion [ ] Stimulate the newborn.

13. How long do you resuscitate a neonate who has asystole and not improving with all measures?

    [ ] 5 minutes [ ] 10 minutes [ ] 15 minutes [ ] 20 minutes [ ] Stop immediately

14. Do you use drugs during resuscitation? [ ] Yes. [ ] No .
    If yes, when? [ ] 1.5 -2 minutes of no response. [ ] Within 3-5 minutes of no response to CPR
    [ ] Between 10 -15 mins of no to response CPR [ ] Immediately [ ] Never

15. How soon do you call for help during resuscitation? [ ] Never [ ] As soon as I identify a baby needs resuscitation [ ] Only when in the theatre [ ] Only in the second stage of labor

Facility related questions

1. Are neonatal resuscitation guidelines available on your ward [ ] Yes. [ ] No

2. Which essential equipment are available in your unit? (*Multiple responses possible)*
[ ] Scissors [ ] Suction bulb [ ] Gloves [ ] Cord Ties [ ] Ambu bag [ ] Clock

[ ] Towels/Cloths [ ] Infant warmer [ ] Polythene bags [ ] NG tubes [ ] Newborn resuscitation table. [ ] None of the above

3. Which of the priority equipment are available in your facility (*Multiple responses possible)*?

[ ] Oxygen concentrator [ ] 100% oxygen [ ] Stethoscope [ ]Suction device

[ ] Continuous Positive Airway Pressure [ ] Pulse oximeter [ ] Transport incubator

[ ] Incubator [ ] Syringes [ ] Glucometer [ ] None of the above

4. Which of the advanced equipment are available in your facility (*Multiple responses possible)*?

[ ] Laryngoscope [ ] Endotracheal tubes [ ]Laryngeal mask airway [ ]Ventilator

[ ] Blood gas analyzer [ ] ECG monitor [ ] None of the above

5. Which of the following drugs are available for use in your facility during resuscitation? (*Multiple responses possible)*

[ ] 10% DW [ ] Normal saline [ ] Adrenaline [ ] Sodium Bicarbonate [ ] None

6. Do you have an equipment check list? [ ] Yes [ ] No.

if yes, how often do you check the list? [ ] Twice daily [ ] Daily [ ] On alternate days [ ]Others, specify___________________

7. Who is responsible for the equipment check?

[ ] Doctor [ ] Nurse [ ] Midwife [ ] No one [ ] Other, specify________________

8. Which of the following are available in your facility? (*Multiple responses possible)*

[ ] Electricity [ ] Backup generator/solar [ ]Ambulance [ ] contact/communication system with referral centres [ ]None of the above
