# Supplementary material for: Facility and care provider emergency preparedness for neonatal resuscitation in Kano, Nigeria
Source: PLoS One. 2022 Jan 7;17(1):e0262446. doi: 10.1371/journal.pone.0262446 (PMC8741031; doi:10.1371/journal.pone.0262446)
Supplement: Attachment — Submitted filename: PLoS ONE preparedness for neo resus Nigeria.docx [file pone.0262446.s002.docx]

The manuscript presents an analysis of provider and facility readiness for neonatal resuscitation in Kano state, Nigeria. The issue examined is very pertinent to address the variable and often slow progress toward global neonatal mortality targets. Limitations in the design of the research restrict its generalizability, though the findings are illustrative of barriers encountered widely.

Abstract: The abstract accurately describes the study. The degree of precision in the percentages reported for adequate knowledge, practice, and facility preparedness could be reduced to whole numbers.

Introduction: It is very pertinent to highlight that Kano State has the highest NMR and thus the highest burden, as the most populous state. It might be useful to characterize the area further (urban/rural, geographic or political/social challenges).

Reference 9 presents a Delphi review that estimated the mortality reduction with neonatal resuscitation to be around 30%. More recent systematic reviews and meta-analyses provide information from actual trials (e.g. Dol J 2018).

While the hypothesis that poor neonatal emergency preparedness contributes to the high prevalence of asphyxia-specific mortality is valid, later discussion of the very low rate of in-facility delivery in the state reveals another major factor contributing to the burden. Addressing this aspect would give a more complete picture of the needs.

Methods: In the description of participants, it should be made clear that this was a convenience sample. Did the selection criteria for attending the workshop skew the participants toward those with low knowledge and skills, or perhaps did it skew toward more experienced clinical leadership?

In the sample size estimation, it is not clear what difference (of 10%) was being measured.

The data collection instrument regarding essential equipment and drugs inquired about several medications that are no longer recommended by ILCOR (International Liaison Committee on Resuscitation) for acute neonatal resuscitation (calcium, bicarbonate, naloxone).

The reliability of data on total number of deliveries and deaths within 24 hours and staff numbers would seem low if based on report from non-supervisory personnel.

What was the basis for considering a facility readiness score of 50% as sufficient to qualify a facility as well-equipped and prepared?

Results: The percentages of respondents could be rounded to whole numbers.

Discussion: When comparing the results to findings of other studies, there is frequent speculation on cause of differences – e.g. “In Ghana….mainly because all respondents were midwives…”. The demographic differences can be highlighted, but causation should not be implied. On page 19, “Western Nigeria reported…..probably because the score criterion….” and “….in Tanzania….probably because the latter study….” should be similarly revised.

The first paragraph of page 20 regarding strengths of the study over-estimates the strength of the design, as it was not a representative sample, nor was self-report validated by observation or triangulation. This paragraph should be revised or omitted.

The role of low utilization of health facilities for delivery should also be highlighted in the discussion. This may be linked to the inadequate knowledge, skills, and equipment in the facilities.

Conclusion: The conclusion appropriately highlights the value of information gained in the study.

Figures and Tables: Figure 2 adds little to the results as presented in the text, and it could be omitted.

References: References are current and complete.
