# Supplementary material for: Facility and care provider emergency preparedness for neonatal resuscitation in Kano, Nigeria
Source: PLoS One. 2022 Jan 7;17(1):e0262446. doi: 10.1371/journal.pone.0262446 (PMC8741031; doi:10.1371/journal.pone.0262446)
Supplement: Attachment — Submitted filename: Response to reviewers.docx [file pone.0262446.s003.docx]

**Responses to reviewers’ comments**

Thank you for considering our manuscript for publication in PLOS One. We have addressed the concerns raised by the reviewers in the table below.

| **Reviewer #1 comments** | **Responses** |
| --- | --- |
| Abstract should be written in sections of introduction, method, result and conclusion. | Done |
| Under abstract it better to define first what mean by emergency preparedness for neonatal resuscitation rather than neonatal resuscitation | Done. The sentence has been modified to fit the context. |
| On line 23, why you proposed an hypothesis “We hypothesize that the high prevalence of asphyxia-related morbidity…”? While your title was preparedness for neonatal resuscitation? This is the same comment for introduction section. | This has been changed to “We hypothesize that poor neonatal emergency preparedness among healthcare providers in Kano, Nigeria may be associated with the dearth of knowledge, poor attitudinal disposition, lack of good practice and equipment shortage at the facility level.” |
| On line 33, include the confidence interval for the result of healthcare provider’s knowledge, skills and facility preparedness | Confidence intervals added. |
| On line 37, what do you mean by weak state and what is your base line to say this? | The basis for this conclusion is from the low level of knowledge, skills and facility preparedness found in our study, which were below the pre-specified reference cut-off values used. |
| On line 55 to 57, you stated that “Even among health workers, the knowledge of neonatal resuscitation is poor, ranging between 2%–12%.[1] In addition, only 8%-22% of surveyed facilities have appropriate resuscitation equipment.[1]”, if these are known, why you study again? | The cited study was done in 6 African countries (Egypt, Ghana, Kenya, Rwanda, Tanzania, and Uganda). It is unclear if findings will be similar in Nigeria, a country with similar demographics that was not included in the cited study. |
| On line 77, you stated as your study will help for policy maker. Do you think that a single site cross sectional study can be influence policy makers? | The study participants were drawn from all the secondary health centres in the state, hence can be described as a multi-centre survey with the potential to inform policy decisions. |
| On line 105 to 109, why you used prevalence of knowledge only for sample calculation, while there are also practices and facility preparedness? Again your sample is different with abstract section (111 Vs 98 and 112 under result section), why this difference? | Two other sample sizes were computed using prevalence of healthcare workers with good resuscitation practices and prevalence of facility preparedness from a similar setting, however, a lower sample size was obtained in both cases. Therefore, the power analysis for prevalence of knowledge was used for this study.  The sample size has been corrected in the abstract to 112. One participant had incomplete records and was excluded from the final analysis, hence, 111 were finally included. In the methods, 98 was the minimum sample calculated. However, all participants that provided consent were included in analysis, which will improve the precision of the findings |
| On line 108, you said that “To increase precision, all healthcare workers who were 109 present at the training were included in the study.”, so why sample calculation is necessary in this case if you were collect data from all them? | Sample calculation was necessary to obtain the minimum sample required to avoid a type II error and for objective extrapolation of results. Including all the 112 survey participants may improve the precision of results and is unlikely to impact negatively on the findings. |
| On line 114, you excluded those who did not provide consent. Do you think that unable to give consent is exclusion criteria? If yes, who were none respondents? | Voluntary participation is an ethical pre-requisite for inclusion. Only one participant declined to take part in the survey. |
| On line 117, you used a semi-structured, self-administered questionnaire. Do you think that it is appropriate method self-administrated questionnaire for knowledge and practice? Why not you used interview administered? | The questionnaire was self-administered to ensure anonymity and reduce response bias. Further, all the participants were literate and could respond to the survey appropriately based on instructions provided. The interviewer to participants ratio was also low, making interviewer administration less efficient and timely. |
| You are using skill, practice and proficiency exchangeable. Therefore, try to use one of it throughout the document | Thank you. Done. |
| Under your analysis section include the p-value you used to select variable for multivariate analysis | P-value used to include independent variables in the multivariate analysis now included |
| Under table 1, check male sex is 405%? | We apologise for the typo. The correct number is 40.5% (now corrected). |
| On line 209, you included attitude. But, there were not stated in the introduction or in method section about attitude. Why this happened/from where attitude came? Even no under operational definition and under measurement. | This information has been added to the abstract, introduction and methods section (outcome variables) to reflect the findings. |
| On line 227, you said that level of practices…, is it a level? If yes, how much level is there? | This phrase has been modified to practice during neonatal resuscitation. |
| In discussion section, try to limit your discussion to your main objectives only. | Thank you. Done |
| Try to copy edit for punctuation (most of your full stop is before your citation bracket in throughout the document). | Thank you. Done |
| **Reviewer #2 comments** |  |
| The abstract accurately describes the study. The degree of precision in the percentages reported for adequate knowledge, practice, and facility preparedness could be reduced to whole numbers. | Done |
| Introduction: It is very pertinent to highlight that Kano State has the highest NMR and thus the highest burden, as the most populous state. It might be useful to characterize the area further (urban/rural, geographic or political/social challenges). | More detail has been added characterizing neonatal mortality in the state based on urban/rural, geographic, or political/social challenges. |
| Reference 9 presents a Delphi review that estimated the mortality reduction with neonatal resuscitation to be around 30%. More recent systematic reviews and meta-analyses provide information from actual trials (e.g. Dol J 2018). | This has been changed to a more recent systematic review. (https://doi.org/10.1136/bmjpo-2017-000183) |
| While the hypothesis that poor neonatal emergency preparedness contributes to the high prevalence of asphyxia-specific mortality is valid, later discussion of the very low rate of in-facility delivery in the state reveals another major factor contributing to the burden. Addressing this aspect would give a more complete picture of the needs. | Done, see lines 468-484 |
| Methods: In the description of participants, it should be made clear that this was a convenience sample. Did the selection criteria for attending the workshop skew the participants toward those with low knowledge and skills, or perhaps did it skew toward more experienced clinical leadership? | Convenience sample has been stated as the method of sampling.  “Using each centre’s work rota, a convenience sample was used for the selection process, inviting only staff that were off duty to eliminate the risk of staff shortage on active duty during the training period. The diversity of the participants including doctors, nurses, and midwives added strength to the sample and reduced selection bias” |
| In the sample size estimation, it is not clear what difference (of 10%) was being measured. | Sample size parameters corrected.  “difference (of 10%)” was a typographical error |
| The data collection instrument regarding essential equipment and drugs inquired about several medications that are no longer recommended by ILCOR (International Liaison Committee on Resuscitation) for acute neonatal resuscitation (calcium, bicarbonate, naloxone). | Calcium and naloxone have been removed. Sodium bicarbonate, however, although discouraged during brief CPR may be useful during prolonged arrest after adequate ventilation is established and there is no response to other therapies. Information regarding its availability in the surveyed facilities to assess level of preparedness may still be useful (DOI: 10.1111/apa.15754 ) |
| The reliability of data on total number of deliveries and deaths within 24 hours and staff numbers would seem low if based on report from non-supervisory personnel. | We agree, but this is a survey looking at participants’ self-reported knowledge, among other things. The findings therefore reflect the perception of the respondents. In the results section, this detail has been revised to reflect self-reported monthly deaths and deliveries |
| What was the basis for considering a facility readiness score of 50% as sufficient to qualify a facility as well-equipped and prepared? | The score was determined using the WHO approach, and facility preparedness indicators identified according to the WHO Service Availability and Readiness Assessment (SARA) Manual (https://apps.who.int/iris/bitstream/handle/10665/149025/WHO_HIS_HSI_2014.5_eng.pdf?sequence=1&isAllowed=y) |
| Results: The percentages of respondents could be rounded to whole numbers. | Done |
| Discussion: When comparing the results to findings of other studies, there is frequent speculation on cause of differences – e.g. “In Ghana….mainly because all respondents were midwives…”. The demographic differences can be highlighted, but causation should not be implied. On page 19, “Western Nigeria reported…..probably because the score criterion….” and “….in Tanzania….probably because the latter study….” should be similarly revised. | Done |
| The first paragraph of page 20 regarding strengths of the study over-estimates the strength of the design, as it was not a representative sample, nor was self-report validated by observation or triangulation. This paragraph should be revised or omitted. | The paragraph has been revised. |
| The role of low utilization of health facilities for delivery should also be highlighted in the discussion. This may be linked to the inadequate knowledge, skills, and equipment in the facilities | Done, see lines 468-484. |
| Figures and Tables: Figure 2 adds little to the results as presented in the text, and it could be omitted. | Figure 2 has now been removed |
| **Reviewer #3** |  |
| - Line 47 “……one-fourth of these deaths…..” revise to …..a quarter of these deaths …. | Done |
| - Line 55 – 57 “Even among health workers, the knowledge of neonatal resuscitation is poor, ranging between 2%–12%.[1] In addition, only 8%-22% of surveyed facilities have appropriate resuscitation equipment”…….please clarify whether these are global or Nigerian statistics | This has been modified to “Reports from 6 African countries showed that even among health workers, the knowledge of neonatal resuscitation was poor, ranging between 2%–12%.[1] In addition, only 8%-22% of the surveyed facilities had appropriate resuscitation equipment.[1] It still remains unclear whether these findings are the same in Nigeria, a country with similar demographics, the most populous in Africa with a high fertility rate, that was not included in the study. |
| Private hospitals were not included as part of the study. Does this mean they do not conduct any births since they were not included in the study? Refer to this related survey about Nigerian health care system at<https://www.shopsplusproject.org/sites/default/files/resources/SHOPS%20Nigeria%20Private%20Sector%20Health%20Census_6.15.2014%20FINAL.pdf> | The focus of our survey were Kano state government-owned secondary health care centres, hence the lack of inclusion of private facilities. |
| Any information on the proportion of births conducted in the three levels of health facilities would be useful to provide the context | Unfortunately, this information is not available |
| How were study participants recruited/informed about the study? | Invitations were sent to participants through the Kano State Ministry of Health Management Board. |
| When and how were the study participants consented to participate in the study? | Participants were informed about the survey at the beginning of the workshop before commencement of the training Written consent was obtained from each participant before inclusion in the study. |
| When was the structured interview questionnaire administered? | The questionnaire was self-administered and completed before the start of the training to ensure efficiency, anonymity and reduce response bias |
| Who collected the data/administered the structured questionnaire? | The questionnaire was self-administered and completed before the start of the training to ensure efficiency, anonymity and reduce response bias. |
| Line 193: Age of participants is better presented by median given the age ranges and distribution | The data for age of the participants was normally distributed, hence our choice of mean as measure of reporting. |
| Line 194 “The majority of the respondents (50.5%, n = 56)…..” this is just half the population and not the majority | This has been changed to “approximately half of the respondents….” |
| It would be useful to know if there were any differences in knowledge/skills scores between participants (66 from secondary hospitals + 45 from paediatric hospitals) | Differences in knowledge and skills scores by type of hospital added. |
| Table 3: Bicarbonate….write the chemical name in full e.g. sodium bicarbonate | Done |
| Page 19, line 307 – 312: “On self-assessed reports of knowledge and facility preparedness, few respondents (8.5% and 32.4%) believed they had a below-average level of knowledge and preparedness, with 90% of the respondents reporting an above-average comfort level during resuscitation. This confirms that the majority of the healthcare providers lack insight into their deficient preparedness both at the individual and facility level, which is a significant barrier for self-motivated learning, improvement and capacity building.” This statement is confusing and unclear. Authors to review and clarify for ease of understanding | This has been reviewed to “Few respondents (9% and 32%, respectively) believed they had a below-average level of knowledge and preparedness. Ninety percent of the participants, however, reported above-average comfort level during resuscitation. This finding, compared with the low participants’ scores on objective assessment of knowledge and practice of NRT confirms that most of the healthcare providers lack insight into their level of individual and facility preparedness for neonatal resuscitation. This is a significant barrier for self-motivated learning, improvement, and capacity building” |
| **Additional remarks** |  |
| Authors are encouraged to further clarify outcome variables and provide the questionnaire used in this study as supplementary file. | Done |
| Authors should explain whether this questionnaire was anonymous or not? | The questionnaire was self-administered and completed anonymously (Lines 243-244 and 328) |
| Authors should provide unidentified raw data of the study. PLOS journals require that authors make all data underlying the findings described in their manuscript fully available, with exception when ethical or legal restrictions prohibit public sharing of data. Authors may upload their data to a public repository or include their data as a Supporting Information file. If data cannot be made public, all restrictions and information necessary for submitting data requests should be outlined in the Data Availability statement, which will be published at the top of the article. | Our data contain potentially identifying and sensitive participant information and therefore cannot be made publicly accessible without permission from the Ethics Review Committee of Kano State Ministry of Health. All data requests should be directed to Mr. Nasir Tafida, Secretary, Research Ethics Review Board, Kano State Ministry of Health, Post office road, Kano Nigeria  Email: [nasitafida438@gmail.com](mailto:nasitafida438@gmail.com)  Phone: +234 (0)8022759125 |
| Authors are encouraged to include and discuss the following relevant and recent publication "Briggs, D. C., & Eneh, A. U. (2020). Preparedness of primary health care workers and audit of primary health centres for newborn resuscitation in Port Harcourt, Rivers State, Southern Nigeria. The Pan African Medical Journal, 36, 68. <https://doi.org/10.11604/pamj.2020.36.68.22164>". | Done, see line 543-545 |
| Please state whether you validated the questionnaire prior to testing on study participants. Please provide details regarding the validation group within the methods section. | Lines 257-259: The final questions used were consensually reviewed, edited, and validated, and were pretested among 12 neonatal health care providers consisting of nurses and doctors in a tertiary institution. |
| In your Data Availability statement, you have not specified where the minimal data set underlying the results described in your manuscript can be found. PLOS defines a study's minimal data set as the underlying data used to reach the conclusions drawn in the manuscript and any additional data required to replicate the reported study findings in their entirety. All PLOS journals require that the minimal data set be made fully available. For more information about our data policy, please see <http://journals.plos.org/plosone/s/data-availability>. | Our data contain potentially identifying and sensitive participant information and therefore cannot be made publicly accessible without permission from the Ethics Review Committee of Kano State Ministry of Health. All data requests should be directed to Mr. Nasir Tafida, Secretary, Research Ethics Review Board, Kano State Ministry of Health, Post office road, Kano Nigeria  Email: [nasirtafida438@gmail.com](mailto:nasirtafida438@gmail.com)  Phone: +234 (0)8022759125 |
