# Supplementary material for: Facility and care provider emergency preparedness for neonatal resuscitation in Kano, Nigeria
Source: PLoS One. 2022 Jan 7;17(1):e0262446. doi: 10.1371/journal.pone.0262446 (PMC8741031; doi:10.1371/journal.pone.0262446)
Supplement: Attachment — Submitted filename: Response to reviewers.docx [file pone.0262446.s004.docx]

**Responses to reviewers’ comments**

Thank you for considering our manuscript for publication in PLOS One. We have addressed the concerns raised by the reviewers in the table below.

| **Reviewers’ comments** | **Responses** |
| --- | --- |
| In the conclusion: “The low level of respondents’ awareness, practice, and facility readiness demonstrates the current weak state of secondary health systems in Kano”. Conclusion has to be based on study findings. Since this study didn’t investigate the “state of secondary health systems in Kano”, authors are encouraged to revise this sentence (reducing its strength/certainty) (e.g., replacing demonstrates by suggests or indicates). | The conclusion has been revised to reflect the study findings and the word demonstrates has been changed to suggest and indicates in the abstract conclusion and main conclusion respectively. |
| In the abstract: “Respondents’ designation predicted good knowledge (aOR=0.08, 95% CI: 0.01–0.69; p= 0.01), but not practice”. Authors should better define/specify “designation” (e.g., physicians versus nurses/midwives). | The designation has been better specified by including physicians versus nurses/midwives to describe it. |
