# Supplementary material for: Facility and care provider emergency preparedness for neonatal resuscitation in Kano, Nigeria
Source: PLoS One. 2022 Jan 7;17(1):e0262446. doi: 10.1371/journal.pone.0262446 (PMC8741031; doi:10.1371/journal.pone.0262446)
Supplement: Attachment — Submitted filename: Response to reviewers copy.docx [file pone.0262446.s005.docx]

**Responses to reviewers’ comments**

Thank you for considering our manuscript for publication in PLOS One. We have addressed the concern raised by the reviewer in the table below.

| **Reviewers’ comments** | **Response** |
| --- | --- |
| Specifically, in the conclusion/results "Respondents’ designation predicted good knowledge (aOR=0.08, 95% CI: 0.01–0.69; p= 0.01). In order to provide the readers with meaningful information, authors should specify "designation" (e.g.,physicians versus nurses/midwives). | The word designation has been changed to profession for clarity and further specified by including physicians versus nurses/midwives to describe it in the abstract, methods, results and conclusion. |
